# DNA Methylation and Schizophrenia: Current Literature and Future Perspective

**DOI:** 10.3390/cells10112890

**Published:** 2021-10-26

**Authors:** Thabo Magwai, Khanyiso Bright Shangase, Fredrick Otieno Oginga, Bonginkosi Chiliza, Thabisile Mpofana, Khethelo Richman Xulu

**Affiliations:** 1 Department of Physiology, School of Laboratory Medicine and Medical Sciences, University of Kwa-Zulu Natal, Durban 4001, South Africa; 210507028@stu.ukzn.ac.za (K.B.S.); 221017678@stu.ukzn.ac.za (F.O.O.); mpofana@ukzn.ac.za (T.M.); 2 National Health Laboratory Service, Department of Chemical Pathology, University of Kwa-Zulu Natal, Durban 4085, South Africa; 3 Department of Psychiatry, Nelson R Mandela School of Medicine, University of Kwa-Zulu Natal, Durban 4001, South Africa; chilizab@ukzn.ac.za

**Keywords:** schizophrenia, DNA methylation, epigenetics, biomarkers

## Abstract

Schizophrenia is a neuropsychiatric disorder characterized by dissociation of thoughts, idea, identity, and emotions. It has no central pathophysiological mechanism and precise diagnostic markers. Despite its high heritability, there are also environmental factors implicated in the development of schizophrenia. Epigenetic factors are thought to mediate the effects of environmental factors in the development of the disorder. Epigenetic modifications like DNA methylation are a risk factor for schizophrenia. Targeted gene approach studies attempted to find candidate gene methylation, but the results are contradictory. Genome-wide methylation studies are insufficient in literature and the available data do not cover different populations like the African populations. The current genome-wide studies have limitations related to the sample and methods used. Studies are required to control for these limitations. Integration of DNA methylation, gene expression, and their effects are important in the understanding of the development of schizophrenia and search for biomarkers. There are currently no precise and functional biomarkers for the disorder. Several epigenetic markers have been reported to be common in functional and peripheral tissue. This makes the peripheral tissue epigenetic changes a surrogate of functional tissue, suggesting common epigenetic alteration can be used as biomarkers of schizophrenia in peripheral tissue.

## 1. Introduction

Neuropsychiatric disorders are heterogeneous disorders that occur as a result of interaction of factors like genetics, epigenetics, neurobiological and environmental factors [1]. These include neurological/neurosurgical disorders and psychiatric disorders [2]. Schizophrenia is also one psychiatric disorder which results in significant socioeconomic burdens [3].

Schizophrenia is a mental illness characterized by dissociation of thoughts, ideas, identity, and emotions [4,5,6,7]. The positive and negative symptoms of schizophrenia result from dysregulated neural pathways in the brain [5,7,8]. Schizophrenia affects approximately 20 million people globally [9]. The life expectancy of individuals with schizophrenia is reduced by 15–20 years compared to the general population [10,11]. This is exacerbated by the coexistence of other disorders like cardiovascular disease (CVD), metabolic syndrome and infectious diseases such as human immunodeficiency syndrome virus (HIV) infection and acquired immunodeficiency syndrome (AIDS) [11,12,13,14]. Understanding the pathophysiology of schizophrenia may lead to better, molecular diagnosis, which may be a key for proper therapeutic interventions. This research is of great interest in countries with large burdens of disease while having several health disparities such as in Africa.

Mental health disorders such as schizophrenia are of great concern in low and middle-income countries (LMIC) like South Africa [15,16,17,18]. Researchers demonstrate that conflicts, hunger, poverty, trauma, social inequality, and poor access to health care in LMIC attribute to the increase of mental health illnesses such as schizophrenia [17,19,20,21]. Neuropsychiatric disorders are the third contributor to the burden of disease in South Africa following HIV/AIDS and other infectious diseases [20,22]. Interestingly, HIV infection is associated with neuropsychiatric disorders like schizophrenia [23]. However, the intersection between these psychiatric disorders and HIV remains poorly investigated.

The diagnosis of schizophrenia has complex heterogeneous clinical syndrome and shares the presentation with several other psychiatric disorders [24,25,26]. Although DSM-5 provides guidelines, schizophrenia diagnosis still remains complex and imprecise [24,27,28]. Both the DSM-5 and the international classification of disease (ICD-11) have incorporated symptom specifiers in the schizophrenia clinical manifestation assessment [28,29,30]. However, the diagnosis of schizophrenia is still complex and there is great need to investigate schizophrenia in order to better understand pathophysiology. Understanding schizophrenia pathophysiology may lead to the next generation of therapeutic drug intervention and molecular biomarkers like epigenetic markers (DNA methylation).

Despite growth in the research of schizophrenia, there is still no clear central pathophysiological mechanism, molecular diagnostic, or precise biomarkers [24,25,26,31]. Epigenetic markers (DNA methylation) hold promise for better understanding of schizophrenia pathophysiology and hope for precise biomarkers. The involvement of different epigenetic markers has been investigated in schizophrenia and there are different approaches that have been undertaken. The current review is on the molecular basis of schizophrenia, with a focus on the epigenetics (DNA methylation) in schizophrenia. The review outlines the different approaches to the study of DNA methylation in schizophrenia, limitations of the current literature, and future perspectives of epigenetics in schizophrenia research.

## 2. The Molecular Basis of Schizophrenia: The Journey So Far

Schizophrenia has an estimated heritability ranging between 79% and 81% [32,33,34]. Many chromosomes harbor a region containing a schizophrenia risk locus [35,36] and many play a role in the development of schizophrenia each with a small to moderate effect sizes [24,37]. Owing to its high heritability and environmental risk factors, schizophrenia is considered a result of gene and environment interaction [38]. Epigenetics links genetics to environmental factors [39].

Dissecting the genetic risk of schizophrenia revealed that there is a polygenic effect in the development of the disease [40,41,42]. Many schizophrenia genetic risk loci are located in the non-coding region such as introns, promoter regions thus, suggesting that gene regulation plays a critical role in the development of the disease [43]. A large number of the schizophrenia risk loci are associated with gene expression [40,44,45], and this implicated epigenetics as a mediator of genetic risk in the pathogenesis of schizophrenia [43].

## 3. The Role of Epigenetic Regulations in Schizophrenia: Gene and Environment Interaction

Epigenetics is the study of genetic alterations directly affecting a gene’s expression without changing the underlying DNA sequence [46,47,48]. Epigenetic mechanisms include DNA methylation, histone modification, chromosomal remodeling, and RNA regulation through non-coding RNAs such as microRNA and long non-coding RNA [49]. Epigenetic markers may shed light to understand better the interaction between genes, the environment, and health-related quantitative traits, such as cognitive function and disease outcomes in schizophrenia [50,51,52,53]. Epigenetic mechanisms regulate the brain’s biology and cognitive process [54]. During the development of the brain, both genetic and epigenetic mechanisms play a crucial role. Hence, if the environment is not suitable for proper genetic response and the epigenetic mechanism is dysregulated, it creates a risk of developing disorders such as schizophrenia [55]. Therefore, epigenetic variations as characterized by altered DNA methylation, histone modification, and microRNA expression or epi-mutation are risks for schizophrenia [56]. Several studies have looked at different epigenetic processes in schizophrenia. However, the current review will only focus on one epigenetic variation, DNA methylation.

## 4. DNA Methylation and Schizophrenia

**Figure 1:**
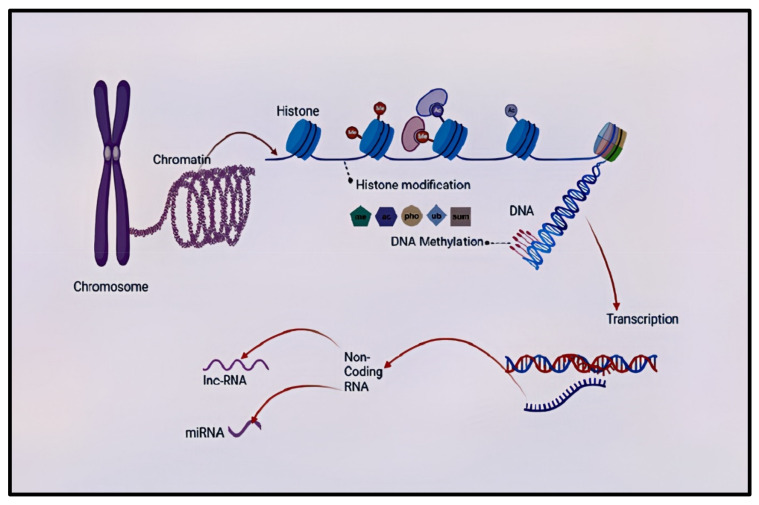
Summary of epigenetic processes that can occur in the mammalian central nervous system. The DNA–protein complex in chromosomes is called a chromatin, who’s functional is a nucleosome (not shown). There is the transcriptionally accessible (found in loosely coiled chromatins) and inaccessible (found in tightly coiled chromatins) DNA. DNA interacts with the N terminal tails of the histone and this gives sites for histone modifications. Histone modification process is a covalent post-translational modification of the histone proteins, and it includes histone methylation (me), acetylation (ac), phosphorylation (pho), ubiquitination (ub), and SUMOylation (sum). Another level of epigenetic regulation is non-coding RNA. DNA methylation occurs when the methyl groups attach covalently to the cyto-sine-guanine dyads (CpG) dinucleotide and non-CpG regions of the DNA. The transcription process makes an RNA copy like mRNA (not shown) from the DNA sequence. Non-coding RNAs can be categorised into long non-coding RNAs and small non-coding RNAs (micro-RNAs). They are involved in chromatin and nuclear remodelling, gene transcription, translational repression, and degradation of messenger RNAs. DNA—deoxyribonucleic acid, RNA—ribonucleic acid, me—methylation, ac—acetylation, pho—phosphorylation, ub—ubiquitination, sum—SUMOylation, miRNA—micro RNA, lnc-RNA—long non-coding RNA.

DNA methylation is the most well-studied epigenetic marker compared to others [57,58]. DNA methylation occurs when the methyl groups attach covalently to the cytosine-guanine dyads (CpG) dinucleotide [59,60]. This methyl group attachment by DNA methyltransferase (DNMT) to forms the 5-methylcytosine (5mC) [61,62,63,64]. The 5mC is then converted by the ten-eleven translocase (TET) protein family to 5-hydroxymethylcytosine (5hmC), which then initiates demethylation. The 5hmC can alternatively be converted by TET to 5-formylcytocine, which can directly be converted to unmethylated cytosine or be converted to 5-carboxycytosine (5CaC). The 5CaC can be removed by base excision repair or be directly converted to unmethylated cytosine (Figure 1) [65]. Emerging research indicates that DNA methylation also occurs at non-CpG sites. DNA methylation can either induce or suppress gene expression depending on other factors and the region where it is situated [66]. To date, several genes have been assessed for DNA methylation as candidate gene for schizophrenia. Table 1 summarizes the some of the studied candidate genes for schizophrenia.

There have been several genes studied as candidate genes for DNA methylation in schizophrenia patients. This review will not cover all the genes but will focus on the genes that are well covered in literature while covering all three pathways, dopaminergic, serotonergic, and GABAergic pathways. Several studies have reported changes in the DNA methylation of different gene in the functional (brain) and/or peripheral (blood) tissues of patients with schizophrenia. This studies have taken several approaches like candidate genes approach, genome wide approach and/or integrated approach. Studies have reported changes in the methylation patterns of several candidate genes. Some of the candidate gene were also reported in genome wide methylation studies. The next sections outline the changes in DNA methylation and supporting evidence from candidate gene, genome-wide and integraded approaches, respectively.

## 5. Gene-Specific/Candidate Gene Methylation in Schizophrenia: Changes in Candidate Gene Methylation in Schizophrenia Patients

### 5.1. Reelin

Reelin (*RELN*) gene encodes an extracellular matrix protein that is involved in cell positioning and neuronal migration during brain development [69,74]. *RELN* has been consistently linked to schizophrenia based on the gene function and the relationship of its variants with schizophrenia [75]. An association has been reported between the hypermethylation of *RELN* promoter and the down-regulation of the expression of *RELN* in the cortical neurons of mice [76,77], in neuroprogenitor NT2 cells [78,79] and the dorsolateral prefrontal cortex of post-mortem tissues [80]. There is contradictory evidence on methylation of *RELN* in the brain and peripheral tissue. Some studies have reported the hypermethylation of the *RELN* gene in the post-mortem brain [80,81] and in peripheral tissue [82] of patients with schizophrenia. However, other studies reported no significant difference in the methylation pattern of *RELN* in the brain [83] and peripheral tissue [84,85] of schizophrenia and controls. The methylation status of the *RELN* gene has been shown to determine the expression of *RELN* in different types of cells like neuronal cells [78].

The methylation of *RELN* gene is thought to most likely be an important factor in the regulation of *RELN* expression and the is also thought to be associated with psychiatric disorders [86]. This is supported by evidence of increased levels of methyl donor S-adenosylmethionine (SAM) in the prefrontal cortex of schizophrenia patients [87] and the association of *RELN* promoter hypermethylation with the *RELN* protein down-regulation [80,88,89,90,91]. A reduction in the levels of *RELN* gene expression and *RELN* protein synthesis induces both dendritic spine density deficits and cognitive impairment in an adult brain [92]

### 5.2. Gamma-Aminobutyric Acid

Glutamate decarboxylase 1 (*GAD1*) also known as glutamate decarboxylase 67 (*GAD67*) gene codes for an enzyme involved in the production of γ-aminobutyric acid (GABA), a major neurotransmitter of inhibitory neurons [67]. Glutamate decarboxylase 1 *(GAD1)* was found to be hypomethylated from the prefrontal cortex of schizophrenia cases [93]. Studies report that the downregulation of Gama-aminobutyric acid (GABA) ergic genes like glutamate decarboxylase 67 (*GAD67*) are mediated by hypermethylation in the frontal cortex and other brain regions. This hypermethylation occurs in the promoter region of the gene [78,80,89,90,94]. This evidence indicates the dysregulated methylation in the promoter regions of genes such as *GAD67* [95].

The *GAD67* promoter hypermethylation has been reported to be associated with the under expression of the gene and protein in patients with schizophrenia [89,90,96]. The altered expression of *GAD67* gene is thought to lead to the impairment of working memory functions and the disturbance in cortical activity and that are evident in schizophrenia patients [97].

### 5.3. Catechol-O-Methyltransferase

The *COMT* gene codes for catechol-O-methyltransferase, which is involved in dopamine metabolism [68,98]. The COMT protein is responsible for the degradation of catecholamine like dopamine and in schizophrenia this process is disrupted [99,100]. There is evidence of an increased methylation in the promoter regions of catechol-O-methyltransferase (*COMT*) [101] in patients with schizophrenia. Furthermore, reports show that there is hypomethylation of membrane-bound catechol-O-methyltransferase *(MB-COMT)* [102,103,104], while soluble catechol-O-methyltransferase *(S-COMT)* is hyper-methylated [105]. However, the hypermethylation of *S-COMT* was not independently confirmed [106]. Other studies have reported increased methylation in the promoter regions of catechol-O-methyltransferase (*COMT*) [101].

An increase in *COMT* methylation is thought to lead a decrease in gene expression [102]. However, the potential risk of schizophrenia is thought to be related to a decreased methylation of the *COMT* gene [102,103,104]. It is thought that a decreased *COMT* methylation in schizophrenia patient leads to an increased expression and thus increased activity of COMT protein. The increased activity then causes low synaptic dopamine levels after the release of neurotransmitters and this eventually lowers dopaminergic stimulation of post-synaptic neurons [107]. When this occurs on prefrontal region (hypofrontility), it may lead to decreased executive functioning commonly seen in schizophrenia patients [108,109].

### 5.4. Brain-Derived Neurotrophic Factor

Brain-derived neurotrophic factor (*BDNF*) is a neurotrophic factor that plays a role in the inflammatory pathway in the central nervous system, and it is considered a candidate gene for the pathogenesis of schizophrenia [70,110]. *BDNF* is a member of the neurotrophic family growth factor supporting differentiation, maturation, and survival of neurons. It shows neuroprotective effects under adverse conditions like glutamatergic stimulation, cerebral ischemia, hypoglycemia, and neurotoxicity [111]. Studies have reported a differential methylation of the *BDNF* gene between patients with schizophrenia compared with controls [85,112,113]. However, other studies could not replicate the results [114,115,116]. This is thought to be due to the presence of cofounders known to affect DNA methylation like age of onset of the disease, gender and use of drugs [117,118]. This makes the standardization of samples and control of cofounders during sampling important.

Aberrant methylation of the *BNDF* gene has been associated with altered expression of the gene [112]. Studies reported an altered *BDNF* expression in the cortical areas of the brain of schizophrenia patients [112,119,120]. This alteration in turn leads to changes in protein levels known to affect dendritic growth, synaptic density, and neuronal cell size [121,122], which are implicated in schizophrenia development.

### 5.5. Sex Determining Region Y-Box Containing Gene 10 (SOX10)

Furthermore, genes outside of the neurotransmitter-related category have also been investigated. Sex-determining region Y (SRY)-box transcription factor 10 (*SOX10)* gene encodes for an oligodendrocyte-specific transcription factor [72]. The *SOX10* gene is responsible oligodendrocyte differentiation [123]. The *SOX10* gene regulates embryonic development and the fate of cells [124]. Iwamoto and colleagues reported the hypermethylation of *SOX10* in the brain of schizophrenia patients and also found that the hypermethylation was correlated to the under expression of *SOX10* [72]. Studies using post-mortem brains of schizophrenia patients have reported that the under-expression of *SOX10* can lead to the dysfunction of oligodendrocytes with the downregulation of the important oligodendrocyte and myelination gene [72,125,126]. Therefore, the CpG DNA methylation status of *SOX10* gene is proposed as an epigenetic sign of oligodendrocyte dysfunction in schizophrenia patients [72].

The expression of the *SOX10* gene is regulated via DNA methylation [72]. Differential methylation of the *SOX10* gene leads to a reduction in the expression of the gene and other oligodendrocyte-related genes [72]. The resultant under expression of *SOX10* then leads to the dysfunction of the oligodendrocytes with the key oligodendrocyte and myelination genes being down-regulated has been reported in schizophrenia patients [125,126,127]. This in turn leads to the downregulation of oligodendrocyte protein evident of schizophrenia [127].

### 5.6. Other Genes

Several previous studies also show differential DNA methylation of genes related to the dopaminergic system, and this is in line with the dopamine hypotheses [128,129,130]. The dopamine theory proposes a hyperactive dopamine transmission in the mesolimbic regions and hypoactive dopamine transmission in the prefrontal cortex of schizophrenia patients [131,132]. Cheng and colleagues [128] have investigated differential methylation in the peripheral blood of participants with schizophrenia. This aforementioned study shows hypermethylation in the promoter region of dopamine receptor D4 *(DRD4)* [128]. Furthermore, Dai and colleagues have found hypermethylation in the promoter of dopamine receptor D3 *(DRD3)* [129]. In another line of evidence, hypomethylation of genes has also been reported. These hypomethylated genes in schizophrenia participants include dopamine receptor D2 *(DRD2)*, *DRD4* and dopamine receptor d6 *(DRD6)* [130]. The promoter methylation of *DRD* genes is thought to alter the expression of the genes and to also be involved in the development of schizophrenia [133]. Insufficient transmission of dopamine in the prefrontal cortex leads to schizophrenia-related cognitive deficits [134,135,136].

Early growth response 1 (*EGR1*) encodes the immediately early protein that belongs to the EGR family of cys-2-his2-type zinc-finger protein [137]. It is implicated in cell proliferation, female reproduction, immune response, cell growth, neutrophil plasticity, and memory formation [138,139]. A decrease in the *EGR1* expression in the peripheral blood and prefrontal cortex of patients with schizophrenia has been reported [140,141,142]. The downregulation of *EGR1* has been reported in the blood and prefrontal cortex of schizophrenia patients [140,142,143]. An upregulation of *EGR1* has been found in the fibroblast and blood of schizophrenia patients [144]. Studies have reported that *EGR1* gene methylation regulates the expression of the *EGR1* gene [73,145]. A decreased *EGR1* gene expression because of *EGR1* methylation has been reported in schizophrenia patients [140,141,142]. This suggests an involvement of *EGR1* gene in the development of schizophrenia [73].

Cholinergic receptor nicotinic alpha 7 (*CHRNA7*) gene encodes the alpha 7 nicotinic acetylcholine receptor (α7-nAChR) which is found on chromosome 15 q13.3, a region that has been identified as a schizophrenia candidate risk locus [71]. The α7-nAChR is a hemopentameric ligand-gated channel with a high permeability for calcium (Ca^2+^) that pre-synaptically increase neurotransmitter release from the specific terminals and post-synaptically affects gene expression [146,147]. *CHRNA7* is considered a promising target for the treatment of cognitive dysfunction [148,149,150]. The *CHRNA7* antagonists have been reported to improve memory and executive function in schizophrenia patients [151]. The promoter methylation of *CHRNA7* gene is thought to lead to a decreased expression of the gene seen in schizophrenia patients [152]. The *CHRNA7* gene is involved in the sensory processing endophenotype seen that is seen in patients with schizophrenic patients [153].However, hypermethylation of genes that regulates serotonin signaling (5-hydroxytryptamine receptor 1A *(5HTR1A)* and serotonin type 2A receptor *(HTR2A)* receptors and serotonin transporter *(5-HTT)* were found to be associated with schizophrenia [154,155,156]. An increase in *5-HT1A* and decrease in *5-HT2A* receptor densities in the dorsolateral prefrontal cortex is associated with both positive and negative symptoms of schizophrenia [157]. Brain-specific angiogenesis inhibitor 1-associated protein 2 *(BAIAP2)* is responsible for dendritic spine density abnormalities [158]. The hypomethylation of *BAIAP2* has been reported in schizophrenia patients [158]. Any reduction in the level of *BAIAP2* are associated with neurological disorder and memory formation deficits [159,160]. *BAIAP2* participates in the proliferation, survival, and maturation of neural cells [161]. 

Parvalbumin (*PVALB*) gene encodes a high affinity calcium ion-binding protein. Deficits of brain parvalbumin (PV) are a consistent finding in schizophrenia [162]. Promoter methylation of the *PVALB* gene has been reported in rats that underwent schizophrenia induction using a sub chronic regime of phencyclidine (PCP) [163]. An increase in the *PVALB* gene promoter methylation was found to be increased in the hippocampus in schizophrenia patients’ post-mortem brain tissue. Since promoter hypermethylation can lead to a reduced gene expression, the reduced expression of PV gene in the brain of schizophrenia patients is thought to be a result of the methylation of the PV gene [162]. 

Peripheral blood showed hypermethylation of cytotoxic T-lymphocyte-associated protein 4 *(CTLA4)*, which is involved in immune function, formation and maintaining peripheral tolerance of T Cells [130]. Promoter and regulatory methylation of the *CTLA4* gene has been reported to alter the expression of the gene [164]. Patients with schizophrenia show alterations in cytokine production and T cell proliferation [165]. The oxytocin receptor gene *(OXTR)* encodes for the oxytocin receptor, which is a key element of the oxytocin system. *OXTR* is hypermethylated in peripheral blood [166]. Differential methylation of *OXTR* is thought to lead to an under-expression of oxytocin receptors [167] and oxytocin is proposed to regulate the central dopaminergic system implicated in the behavioural manifestations of schizophrenia [168,169]. 

Initial DNA methylation studies focused on DNA methylation alterations in candidate genes. The results from candidate gene studies are conflicted. The candidate gene approach was limited by its coverage of the genome and as such authors resorted to genome wide DNA methylation approach for a more comprehensive coverage.

## 6. Genome-Wide Methylation Studies in Schizophrenia: Evidence of Changes in DNA Methylation in Schizophrenia Patient

Genome-wide investigations to determine variations on methylation to provide an insight into mental health disorders like schizophrenia is insufficiently in the literature despite emerging data [170]. The first genome-wide DNA methylation study reported significant epigenetic changes associated with schizophrenia land bipolar disorder in the prefrontal cortex of patients with major psychosis using a microarray [171]. This was subsequently confirmed by Dempster and colleagues in the blood samples from 22 twin pairs discordant for SC and BP using microarray [172]. Following that, there was an increase in genome wide DNA methylation studies using different methods.

Genome-wide methylation studies by nature cover a wide range of differentially methylated patterns and due to that, they are known to have a complication with replication of results. There has been a problem in replicating the results of genome-wide studies. Despite this, there have been some common methylation patterns that were noted with genome-wide studies. The glutamate ionotropic receptor alpha-amino-3-hydroxy-5-methyl-4-isoxazole propionate (AMPA) type subunit 1 (*GRIA1*) gene methylation was reported by two different studies [171,173]. The *GRIA1* gene codes for one of the four ionotropic AMPA receptor subunits and is involved in synaptic plasticity [174]. Some of the genes reported by genome-wide studies are the known candidate genes for schizophrenia. These genes include *RELN* [175,176], *COMT* [176], *DTNBP1* [177] and *SOX10* [177].

Genome-wide investigations to determine variations on methylation to provide an insight into mental health disorders like schizophrenia are insufficiently reported in the literature despite emerging data [170]. Also, different populations of different demography are not yet covered, especially African populations. Furthermore, most mental health conditions like schizophrenia consist of the polygenetic effect of pleiotropic genes. Therefore, precise etiology to infer specific molecular basis like genetic or epigenetic variations is lacking [170]. To further understand the molecular basis of schizophrenia, it is essential to investigate different aspects of the molecular basis of the disease, from genes to their regulations such as epigenetics and their expression [178,179,180,181]. This line of research is critical as it may provide a direction for precision medicine for mental health disorders like schizophrenia. Therefore, genome-wide epigenetics and gene expression may shed light to understand the diseases better as well as provide insight on prognosis, diagnosis, and treatment outcomes. 

Despite the use of genome-wide methylation studies, there was still a problem with identifying consistent schizophrenia specific DNA methylation patterns. This is due to the lack of reproducibility of results in genome-wide studies. This has been attributed to several limitations of studies that have been conducted thus far. Most of the genome-wide methylation studies used methylation arrays which are by design limited to the already known DMR and as such will not cover the entire genome. Most of the arrays only cover CpG regions and it has been noted that non-CpG methylation occurs. Some of the studies also did not control for the cell heterogeneity, age of diagnosis, cause of death in post-mortem brains, use of medication, and age of patients.

## 7. Integrated DNA Methylation and Gene Expression: Multi-Omic Approach—Towards Precision Medicine

Based on the current data from genome-wide association studies and epigenome-wide association studies, it has become more apparent that a single omics approach might not be adequate in the search for mental health disorder development and potential biomarker discovery. This makes multi-omics/integrated studies of utmost importance in the quest to understand mental health disorders.

Zhu and colleagues reported that the DNA methylation to be correlated with gene expression in the peripheral blood of monocytes of monozygotic discordant twins [182]. Another intergraded study using whole blood DNA methylation and gene expression data from the Gene Expression Omnibus database reported the identification of blood-based signature with 46 hypo-up and 71 hyper-down genes and the authors posits that the genes may have a potential role in the diagnosis of major depressive disorder [183]. Another study by van Eijk and colleagues identified 1095 differentially methylated regions associated with 1226 differentially expressed genes. They also reported that half of the transcripts were showing differential expression in the peripheral blood of schizophrenia patients [184]. 

Integration of DNA methylation, gene expression, and the consideration of its global effects are essential in the understanding of the mechanism with which DNA methylation leads to the development of schizophrenia [185]. A significant increase in the expression of miRNAs has been reported in schizophrenia patients [186]. 

A cell-type-specific study integrating the methylome and transcriptome of distinct cells from post-mortem brain tissues showed the importance of cell and tissue type epigenetics and the importance of a whole-genome approach [43]. The study was however limited using post-mortem human brain tissue due to the cofounders related to the use of such tissues [43,187,188]. Literature also shows that the bulk of the studies on epigenetics of schizophrenia and psychosis suffer from methodological limitations. Most of the studies used pre-defined microarrays, which traditionally doesn’t cover the whole genome thus making an unbiased, whole-genome DNA methylation approach covering methylation even outside promoter region and CpG island very important in the understanding of the role of Epigenetics in mental disorders [43,189,190].

Most of the studies looking into the involvement of non-coding RNA (ncRNAs) in schizophrenia focused on microRNAs other than other ncRNAs [185,191,192]. The expression of miR137 has been reported to be altered in schizophrenia [42,193,194]. miR137 expression is essential in several signaling nodes in several gene networks that are relevant to the development and function of the brain [195,196]. 

An integrative study reported an overlap of intronic deferentially methylated region with miRNA. The study also reported that the aberrant DNA methylation-related miRNAs were differentially expressed thus suggesting that DNA methylation may be affecting gene expression and eventual protein expression via miRNAs [185]. 

Several studies have profiled the expression of miRNAs in the blood of schizophrenia patients to discover blood-based biomarkers for schizophrenia [197,198]. A recent genome-wide expression study reported that several miRs were differentially expressed in the serum of schizophrenia patients. The study reported 11 miRs that could differentiate the schizophrenia patients from controls [197]. 

More studies are required that intergrade different omics. Multi-omics studies will generate valuable data in the understanding of the pathophysiology of schizophrenia and possible search for biomarkers. The studies are required to account for the limitation seen in current literature. There are different limitation and cofounders seen in the current studies of DNA methylation in schizophrenia. The next section outlines the limitation and cofounders and their influence of DNA methylation. The next section also outlines the use of alternative models of schizophrenia, like animal model, in addressing limitation and controlling for cofounders.

## 8. Limitations of Previous Studies and Future Perspectives: Where to from Here?

Several studies have been completed in search of the potential role of epigenetics in the development of schizophrenia. The studies have yielded a lot of potential data in this regard but there is a problem with reproducibility [170]. This has been attributed to several limitations of studies that have been conducted thus far. The limitations are small sample size, the type of sample used for analysis [67], use of medication [118], smoking [117], tissue and cell-type heterogeneity [199] and methods of studying the epigenetics [54]. 

Epigenetics studies have widely been performed using post-mortem brain [171,177,200] and peripheral blood samples [103,173,175] this is thought to be due to the relative ease in obtaining these samples compared to brain tissue from live patients. The use of these samples introduces cofounders to the study like smoking tobacco, alcohol use, disease course, use of medication, age, infections, time, and cause of death [117,118]. Also, the epigenetic profile of the brain and peripheral tissue is different [201] and schizophrenia is a disorder of the brain [202]. All the above cofounders have been shown to influence the epigenome and transcriptome [124]. Although some of the cofounder like smoking has been shown to alter global DNA methylation [203], the control of these cofounders is required for a clear picture of the effect of methylation on schizophrenia. The use of alternative samples like samples from animal models or induced cell models may assist in the control of these cofounders.

One of the limitations is the use of medications. Studies have shown that antipsychotics affect the patterns of DNA methylation [204,205,206,207]. Olanzapine has been found to change DNA methylation patterns of the brains of mice [207], and clozapine was found to change DNA methylation in human peripheral leukocyte [208]. Studies have reported that antipsychotics may affect the promoter methylation of genes related to schizophrenia [206,207]. Antipsychotic drugs can alter the methylation patterns of schizophrenia genes and related gene expression, however, certain methylation patterns prior to the use of antipsychotics can affect the influence the efficacy of antipsychotics [105,209]. Studies are required to account for the use of medication as medication can alter the methylation patterns. However, with the use of post-mortem brain tissue, the control for use of medication is limited. This brings make the use of animal and cell model for schizophrenia of importance as they present a chance for treatment naïve schizophrenia model.

Another major limitation in previous studies is the tissue and cell-type heterogeneity in samples used for analysis [199]. It has been reported that different cell types have different epigenetic patterns [210,211,212]. There are considerations regarding the stability and biological implications of the epigenetic measurements in post-mortem tissue as changes in DNA methylation have been reported with post-mortem interval [43,187,188]. The use of mixed tissue and cell type samples limits the identification of epigenetic and transcriptomic changes in schizophrenia as the changes are masked by changes in other tissue and cell types [124]. Studies are required to control for cell and tissue type heterogeneity in the sampling process to give a clear picture of DNA methylation in different parts and cell of the brain schizophrenia. This will help with the pathophysiology of schizophrenia and with evidence needed for the development of new therapeutic targets and diagnostic markers.

Despite the different approaches used, there still is limited reproducibility and this can also be attributed to the method used to assess DNA methylation in different studies [54]. The methodological limitations are related to the different coverages of the available methods. Targeted approaches are by definition limited in their coverage and will only pick up methylation in targeted genes [213]. To deal with this limitation, genome-wide approaches are used as they cover the whole genome methylation [171,172]. This however is also limited based on the coverage of the platform used. Most genome-wide studies are using methylation arrays, which use predefined probes for the detection of methylation, which is biased in their analysis of methylation [43,189,190]. An unbiased genome-wide methylation approach has revealed methylation outside the promoter regions and non-CpG methylation are important [43]. For an unbiased picture of DNA methylation in schizophrenia patients, studies are required to look at CpG and non-CpG genome wide methylation. Such studies will provide a holistic picture thus enabling further understanding of the pathophysiology of schizophrenia and assisting in the search for diagnostic biomarkers. 

To understand the potential involvement of the epigenome in the development of schizophrenia, studies that control for the above-mentioned limitation are needed. The studies need to control for, in sampling, the sample type, tissue, and cell-type heterogeneity. Studies should also use methods that have a broader coverage like sequencing [43]. To complete the search for potential biomarkers for schizophrenia, studies with defined tissue and cell types should compare differentially methylated region in brain tissue and peripheral blood tissue as the common epigenetic pattern in both brain and blood could serve as a potential biomarker [140,177,214].

Data from schizophrenia animal models shows added evidence of the effect of environmental factors on epigenetic [215]. Different animal models have been used and are based on factors implicated in schizophrenia like maternal immune activation during pregnancy, inhibitors of foetal neurogenesis, pre- and post-natal stress, nutritional deficiencies, drug abuse, exposure to toxicants, reduced postpartum maternal care, and cannabis use in adolescence. The models capture a wide spectrum of neurological and behavioural changes related to schizophrenia and shows difference in several epigenetic markers [124,216]. The use of schizophrenia animal models enables the comparison of epigenetic markers like DNA methylation in functional and peripheral tissues and different stages of development [213] and testing target sequences that are influenced by changes in the chromatin and to also test their effects on the development and functioning of the brain [217]. The use of animal models enables researchers to study mental health using samples that are free from cofounders like use of medication, smoking, alcohol use, cause of death, infections, cannabis use and disease course.

Several limitations have been noted with current studies and new studies are required to control for these limitations. The use of animal models for schizophrenia is of importance in addressing some of the limitations. Control of cofounders like age, use of medications, smoking can be relatively challenging with the use of post-mortem brain tissue. The use of animal models is necessary for the control of these cofounders. 

## 9. Possibility of Blood-Based Biomarker: Towards a Laboratory Screening, Diagnosis, and/or Monitoring Tool

A biological marker, or biomarker, is a trait that can be evaluated and measured as an indicator of biological process, pathological process, or treatment response [218]. There are currently no precise and functional biomarkers for neuropsychiatric disorders like schizophrenia [219]. Studies of epigenetic changes in schizophrenia hold promise for the development of diagnostic and prognostic biomarkers for schizophrenia and a therapeutic target [177]. To achieve this goal, studies are required that will identify aberrant DNA methylation profiles in functional tissue and determine if the results are translatable to diagnostically feasible tissue [177].

Methylation status changes in peripheral tissue like blood are thought to mirror methylation changes in the brain [220]. Some methylation markers were found to be similarly altered in both brain and peripheral tissue. Several studies have attempted to compare the methylation patterns among brain and peripheral tissue and these studies identified about 2–7% of CpG sites that showed a correlation between the brain and peripheral tissue [201,221,222,223]. This suggests that such common epigenetic alterations may be used as potential biomarkers for schizophrenia. Peripheral epigenetics are important in the identification of biomarkers, but the epigenetics signature of the brain isn’t a mirror image of the peripheral tissue. This means that common changes must be found and replicated between the brain and peripheral tissue.

Memories are stored at the molecular level and in the functional organization of the brain and mind, combining to alter cognition, behavior, and clinical symptoms [224,225]. Since all memories are controlled by molecular mechanisms, they require gene expression changes and protein synthesis. These proteins are encoded by genes which can be regulated via epigenetic modification [226,227,228]. The diagnostic procedure of schizophrenia is difficult, and this is because it’s a heterogeneous clinical syndrome [24,27]. Although the current diagnosis incorporated symptom specifiers [28,29,30], the diagnosis of schizophrenia is still difficult and imprecise [24,27]. Thus, making it necessary to discover new, better ways, including biomarkers for diagnosis of schizophrenia. Studies are required that will compare DNA methylation in functional tissue with peripheral tissues to assist in the search for biomarkers.

## 10. Conclusions

Based on the results and limitations from the current literature, we envisage the need for a tissue and cell type specific, unbiased whole genome DNA methylation study integrated with gene expression, considering global effects of DNA methylation (miRNAs). The study will need to control for confounders related to the use of post-mortem tissue and compare the epigenetics of the CNS tissue and non-CNS tissue for biomarker discovery. Peripheral epigenetics are important in the identification of biomarkers, but the epigenetics signature of the brain is not a mirror image of the peripheral tissue. This means that the common changes must be found and replicated between brain and peripheral tissue.

## Figures and Tables

**Table 1:** Summary of some of the candidate gene methylations for schizophrenia.

Candidate Gene	Gene Location	Encoded Protein	Major Function	Reference
*GAD1*	2q31.1	Glutamate decarboxylase-67	Conversion of glutamic acid to GABA	[67]
*COMT*	22q11	Catechol-O-methyltransferase	Monoamine metabolism	[68]
*RELN*	7q22	Reelin	Cellular maturation and synaptic function	[69]
*BDNF*	11p13–14	Brain Derived Neurotrophic Factor	Survival and differentiation of neuronal population	[70]
*CHRNA7*	15q13.3	Nicotinic acetylcholine receptor	Cholinergic synaptic transmission	[71]
*SOX10*	22q13.1	SRY-box 10	Nucleocytoplasmic shuttle in the development nervous system	[72]
*EGR1*	5q31.2	Early growth response protein 1	Transcriptional regulator in neuronal development	[73]

GABA-gamma aminobutyric acid, SRY-sex determining region Y.
